# The Impact of Whole-Body Cryotherapy on Endothelium Parameters in Patients with Ankylosing Spondylitis

**DOI:** 10.3390/antiox12020521

**Published:** 2023-02-19

**Authors:** Agata Stanek, Ewa Romuk, Tomasz Wielkoszyński, Klaudia Brożyna-Tkaczyk, Daria Wziątek-Kuczmik, Armand Cholewka

**Affiliations:** 1 Department and Clinic of Internal Medicine, Angiology and Physical Medicine, Faculty of Medical Sciences in Zabrze, Medical University of Silesia, Batorego 15 St., 41-902 Bytom, Poland; 2 Department of Biochemistry, Faculty of Medical Sciences in Zabrze, Medical University of Silesia, Jordana 19 St., 41-808 Zabrze, Poland; 3 Social and Medical Faculty, Higher School of Strategic Planning, Kościelna 6 St., 41-303 Dąbrowa Górnicza, Poland; 4 Chair and Department of Internal Medicine, Medical University of Lublin, Staszica 16 St., 20-081 Lublin, Poland; 5 1st Department of Cranio-Maxillofacial Surgery, Faculty of Medical Sciences, Medical University of Silesia, Francuska20/24 St., 40-027 Katowice, Poland; 6 Faculty of Science and Technology, University of Silesia, Bankowa 14 St., 40-007 Katowice, Poland

**Keywords:** whole-body cryotherapy, ankylosing spondylitis, endothelium parameters, oxidative stress

## Abstract

Background: The aim of the study was to assess the effect of whole-body cryotherapy (WBC) with subsequent exercise training (WBC group) or exercise-only training (ET group) on endothelium inflammation parameters in patients with ankylosing spondylitis (AS). Methods: The WBC procedure lasted 3 min, and exercise training consisted of one 60 min session a day, which was the same in each group. The ET group was compared to the WBC group. Endothelium (high-sensitivity C-reactive protein (hsCRP), soluble P-Selectin, soluble vascular cell adhesion molecule-1 (sVCAM-1), neopterin), and oxidative stress (lipid hydroperoxide (LHP), protein sulfhydryl (PSH), lipofuscin, paraoxonase-1(PON-1), and albumin) parameters were estimated 1 day before and 1 day after the completion of the study. Results: A significant decrease in hsCRP, sP-Selectin, sVCAM-1, and neopterin concentrations was observed in the WBC group after the treatment. After the treatment, in both groups, LHP and lipofuscin levels and PON-1 activity decreased significantly. The observed drop in these parameters was higher in the WBC group compared to the ET group. Albumin concentration increased in the WBC group after treatment. Conclusion: Procedures of WBC have a beneficial effect on endothelium parameters in AS patients; therefore, this method can be applied in the treatment of this group of patients.

## 1. Introduction

Ankylosing spondylitis (AS), a type of spondyloarthropathy (SpA), is characterized as a chronic inflammatory rheumatic disease, in which macrophages play a major role in the inflammation process. The first symptoms affect the spine, sacroiliac joints, and entheses [1]. The inflammatory process leads to fibrosis and calcification, which results in loss of flexibility and the fusion of the spine [2]. Inflammatory back pain and stiffness, with syndesmophytes and spinal ankylosis, are the most characteristic symptoms of the disease. Decreased quality of life and reduced physical functions are caused by ossification of the spine [3]. In addition, inflammation of the skin, inflammatory bowel diseases, enthesitis, and anterior uveitis may occur [4].

The prevalence of AS is estimated to be 0.03–1.8% in Europe, North America, and China [5]. The disease is around three times more common among men than women [6]. Patients with AS have been shown to have increased cardiovascular (CV) mortality, twice the incidence of ischemic heart disease than the control group alone, and an elevated prevalence of traditional CV risk factors than the general population [7]. In addition, in patients with AS, the risk of myocardial infarction rises in younger patients and those with a more serious disease, determined by disease activity. This was observed even after modifying the traditional cardiovascular risk factors such as hyperlipidemia, hypertension, diabetes, smoking, and body mass index [8]. A systemic inflammatory pattern and increased oxidative stress are key points of endothelium dysfunction and accelerated atherosclerosis in this group of patients [9,10]. In addition, AS patients without known traditional cardiovascular risk factors have been reported to have increased carotid intima-media complex thickness, adhesion molecule levels, oxidative stress, inflammation, lipid profile parameters, and serum levels of soluble CD40 ligand (sCD40L) compared to matched healthy individuals. These factors may enhance the development of atherosclerosis in AS patients [9,11,12].

According to ASAS-EULAR (European League Against Rheumatism) recommendations from 2016 regarding the treatment of AS, it is recommended to combine nonpharmacological and pharmacological treatment in order to reduce patient discomfort [13]. Exercise and physiotherapy are considered fundamental tools for the management of AS patients [14]. Supervised physiotherapy gives better results than usual care, which is manifested in decreasing disease activity and pain, as well as improving functional capacity in patients with AS [15]. It seems that a combined exercise program (range of motion, strengthening, and aerobic exercises) brings more benefits than a range of motion exercise alone [16].

Whole-body cryotherapy (WBC) is one of the physiotherapeutic methods used in patients with rheumatic diseases, in which the human body is exposed to very low temperatures (below −100 °C) for 120–180 s [17,18]. Immediately after WBC procedures, patients usually undergo exercises. Due to WBC, exercise intensification and extension of its duration are possible. The combination of WBC and therapeutic exercises are key elements of cryorehabilitation [19,20].

So far, WBC procedures have shown that, in patients with AS, they can reduce oxidative stress, inflammatory, and lipid profile parameters [21,22,23], as well as improve some spine mobility and decrease pain [19,20]. In our previous research [24], it was shown that, in healthy subjects, whole-body cryostimulation may have a beneficial impact on endothelium inflammatory parameters. So far, there are no publications on this topic in patients with AS. In light of the above findings, we would like to study the influence of WBC on some endothelium parameters in patients with the active phase of AS and without concomitant cardiovascular risk factors.

## 2. Materials and Methods

The study was carried out according to the Declaration of Helsinki and approved by the Bioethical Committee of the Medical University of Silesia in Katowice (permissions no. NN-013-149/I/02 and NN-6501-93/I/07, Poland). All patients signed the consent form for inclusion in the study. The patients were informed about the purpose and course of the study, the possibility of withdrawing from participation in the project at any stage, and access to insight into their results. Detailed information on the behavior in the cryochamber and the required clothing was given.

### 2.1. Patients

The protocol for qualifying patients for the study was similar to those of our earlier studies [19,21,22]. A total of 32 nonsmoking male patients with ankylosing spondylitis who had never been subjected to any form of cryotherapy were involved in the study. The patients were divided randomly into two groups with an allocation ratio of 1:1, i.e., 16 AS patients exposed to WBC with exercise training (WBC group, mean age 46.63 ± 1.5 years) and 16 AS patients exposed to exercise training group (ET group, mean age 45.94 ± 1.24 years). There were no significant differences in the mean age, body mass index (BMI), Bath Ankylosing Spondylitis Diseases Activity Index (BASDAI), Bath Ankylosing Spondylitis Functional Index (BASFI), and comorbidities and classical cardiovascular risk factors between groups. 

Patients with AS were treated with nonsteroidal anti-inflammatory drugs (NSAIDs). The doses of drugs were not changed within the month before and during the study. Patients involved in the research fulfilled the modified New York Criteria for definite diagnosis of AS, which serves as the basis for the ASAS/EULAR recommendations [25]. Ultimately, we selected only HLA B27-positive patients, who exhibited II and III radiographic grades of sacroiliac joint disease and stayed in the active phase of the disease. Exclusion criteria were as follows: the presence of contraindications for whole-body cryotherapy treatments, exercise training, the usage of vitamins, hormones, supplements, immunomodulators, and immunostimulators for 4 weeks prior to the study, smoking, any other comorbidities, and treatment with disease-modifying antirheumatic drugs (DMARDs), biologic agents, or steroids. The inclusion/exclusion criteria for the ET (control) group were the same as for the WBC group. Table 1 includes the demographic data of the patients. Before laboratory analyses, the patients were asked to refrain from consuming caffeine for 12 h. The patients’ diet was not modified during the study. For safety reasons, before the study, all patients were examined by a physician, were subjected to a resting electrocardiogram, and had their blood pressure measured before each cryotherapy session.

### 2.2. Whole-Body Cryotherapy and Exercise Training Protocol

In the study, we used the WBC scheme and exercise training based on our previous experiences [19,21,22]. The AS patients underwent ten WBC treatments in a Wroclawski-type cryochamber (Creator, Poland). It was cooled with liquid nitrogen. The concentration of oxygen in the air of the cryochamber was kept constant at 21–22% and continuously controlled. The WBC procedure included five treatments a week, from Monday to Friday, in the morning for 2 weeks. Each procedure started with a 30 s stay in the vestibule at −60 °C and then continued at about −120 °C for 3 min. During the procedure, patients moved slowly in a circle and changed the direction of walking every 30 s. They were also instructed to breathe slowly. The treatments took place under the supervision of a trained employee working in the facility. At any time, it was possible to interrupt the procedure, for both the supervisor and the patient. During WBC treatment, patients were dressed in shorts, woolen knee socks, gloves, headbands or caps covering the auricles, and clogs. The nose and mouth were covered with a surgical mask. Before entering the cryochamber, patients thoroughly dried their skin of sweat and, if necessary, removed jewelry, watches, and contact lenses.

**Figure 1:**
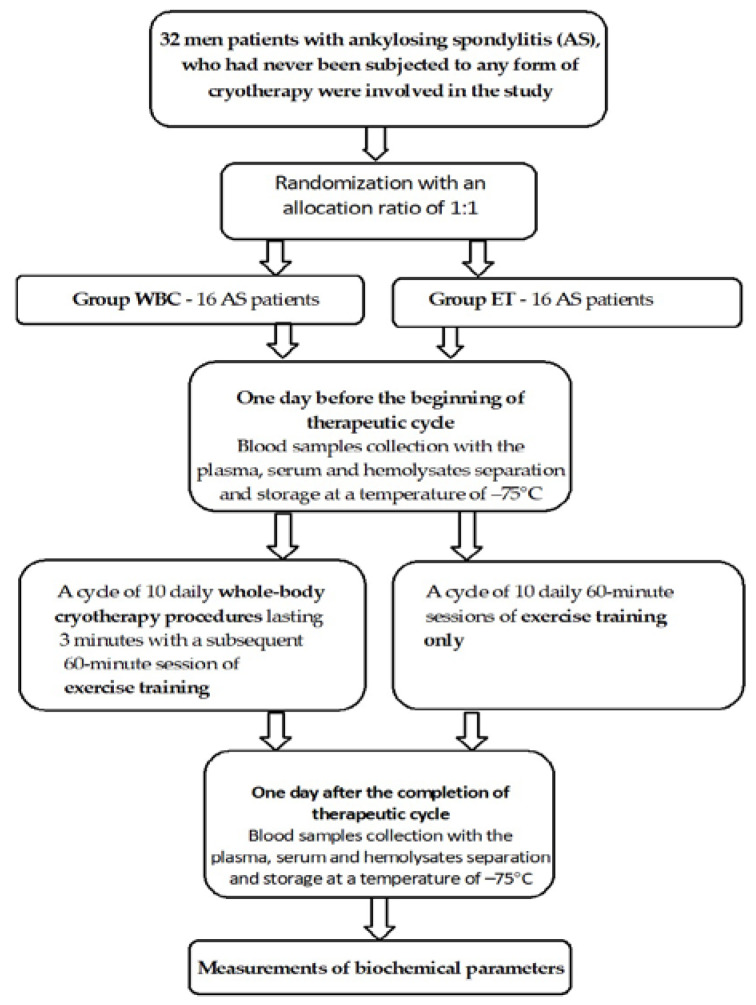
Scheme of study protocol.

Immediately after exiting the cryochamber, AS patients started 1 h of exercise training. The exercise program was the same for all patients. Physical training involved exercises targeting the range of motion of the spine and major joints (including hip, knee, ankle, shoulder, elbow and wrist). Chest expansion and breathing exercises were also applied. In addition to physical exercise, patients with AS were exposed to exercises strengthening the muscles of the main parts of the body (spine, arms, and thighs) and aerobic exercises (including cycling and brisk walking). All exercises were performed in the presence of physiotherapists [16,19]. A scheme of the study protocol is presented in Figure 1. 

### 2.3. Biochemical analysis

All measurements (endothelium function and oxidative parameters) were performed on all 16 patients in each group. Blood samples were collected in the morning before the first meal, once the day prior to any intervention and the a second time the day after the end of the treatment period. Fasting blood samples were collected from the basilic vein using the S-Monvette system (Sarstedt) into tubes with EDTA-K3 (1.6 mg/mL; 2.7 mL whole blood) and tubes with clot activator (2.7 mL of blood). Next, samples were centrifuged (10 min, 900 g at 4 °C), and EDTA-plasma and serum were immediately aliquoted and stored at −75 °C for future biochemical analysis. Then, the remaining erythrocytes in the EDTA tubes were rinsed three times with phosphate-buffered saline (pH 7.4). After final centrifugation, erythrocytes were lysed in 10 mM TRIS/HCl buffer (pH 7.4). The concentration of hemoglobin in the hemolysates was determined using Drabkin’s reagent. The repeatability and reproducibility of the cyanmethemoglobin method were 1.4% and 2.6%, respectively.

#### 2.3.1. Endothelium Function Parameters

The concentration of high-sensitivity C-reactive protein (hs-CRP) in the serum was examined using the latex immunoturbidimetric method (BioSystems, Spain) and expressed in mg/L. The inter- and within-test coefficients of variation (CV) were 2.4% and 5.6%, respectively.

Soluble P-Selectin (sP-Selectin) in plasma was measured with the use of a commercially available kit (sP-Selectin ELISA, BioVendor, Brno, Czech Republic). The minimal detectable concentration was approximately 0.2 ng/mL. The concentration of sP-Selectin was expressed as ng/mL. The test range was 0.63–40 ng/mL. The inter- and within-test coefficients of variation (CV) were 5.4% and 7.8%, respectively. This ELISA was performed using a BioTek Elx800 reader (BioTek Instruments Inc, Tecan Group, Switzerland). 

Soluble vascular cell adhesion molecule-1 (sVCAM-1) in plasma was measured with the use of a commercially available kit (sVCAM-1 ELISA, BioVendor, Brno, Czech Republic). The sVCAM-1 concentration was expressed as ng/mL. The assay sensitivity was 0.6 ng/mL. The range of the assay was 3.15–100 ng/mL. The inter- and intra-assay coefficients of variations (CV) were 3.1% and 5.2%, respectively. This ELISA test was performed with the use of a BioTek Elx800 reader (BioTek Instruments Inc, Tecan Group, Switzerland). 

Neopterin in serum was measured with the use of a commercially available kit (Neopterin ELISA, Wuhan Fine Biotech, Wuhan, Hubei, China). The neopterin concentration was expressed as ng/mL. The assay sensitivity was 0.094 ng/mL, and the assay range was 0.156–10 ng/mL. The inter- and intra-assay coefficients of variations (CV) were 8% and 10%, respectively. This ELISA test was performed with the use of a BioTek Elx800 reader (BioTek Instruments Inc, Tecan Group, Switzerland).

#### 2.3.2. Oxidative Stress Parameters

The concentration of lipid hydroperoxide (LHP) in serum was measured in accordance with Södergrena et al. [26] using xylenol orange. In this procedure, iron(II) ions are oxidized to iron(III) in an acidic environment. The formed iron(III) gives a blue-purple complex with xylenol orange. The concentration was read at a wavelength of 560 nm using a Victor-X3 reader (PekinElmer) from a calibration curve prepared using appropriate concentrations of H_2_O_2_. LHP concentration was expressed in μmol/L.

In erythrocytes, the lipofuscin concentration was measured using the method of Jain [27]. Extraction was performed with use a 2-propanol/chloroform mixture added to the erythrocytes in a ratio of 3:2 (*v*/*v*). After centrifugation, the clear supernatant was measured with the use of an LS45 spectrofluorometer Perkin Elmer at 360 nm (excitation) and 440 nm (emission). The lipofuscin concentration was expressed in relative units (relative lipid extract fluorescence, RF), where an RF value of 100 corresponds to the fluorescence of a solution of 0.1 mg/mL quinine sulfate in 0.05 M sulfuric acid. LPS concentration is shown as RF per gram of hemoglobin (RF/g Hb). The inter- and intra-assay coefficients of variations (CV) were 5.8% and 7.4%, respectively. 

Koster’s method, using dithionitrobenzoic acid (DTNB), was used to determine the serum concentration of protein sulfhydryl (PSH) [28]. The concentration of sulfhydryl groups was expressed in µmol/L and was calculated from a calibration curve using reduced glutathione as a standard. The inter- and intra-assay coefficients of variations (CV) were 2.5% and 5.3%, respectively.

Serum paraoxonase-1 (PON-1) activity was assayed spectrophotometrically with the use of paraoxon (o,o-diethyl-o-(*p*-nitrophenyl)-phosphate) as a substrate [29]. Determinations were performed on a BM250 biochemical autoanalyzer (Emapol, Poland) at 405 nm and 37 °C with kinetic mode. The repeatability and reproducibility of the method were 2.7% and 4.5%, respectively.

The serum albumin level was measured using a commercially available ELISA kit (Albumin ELISA, BioVendor, Brno, Czech Republic). The assay sensitivity was 39 pg/mL, and the assay range was 0.78–50 ng/mL. This ELISA test was performed with the use of a BioTek Elx800 reader (BioTek Instruments Inc, Tecan Group, Switzerland). The serum albumin level was expressed as g/L. Inter- and intra-assay coefficients of variations (CV) were 5.1% and 6.8%, respectively.

### 2.4. Statistical analyses

Indices were presented as the mean (x) and standard deviation (SD). Two independent groups of patients (WBC and ET) were checked before and after therapy using repeated measures. However, this required a verification of the normality and homogeneity of variance, followed by the Student’s t-test or Wilcoxon’s test. To compare differences between groups, an independent-sample Student’s t-test or the Mann–Whitney U test was used. Differences were considered statistically significant for a *p*-value <0.05. The statistical package of the Statistica 10 Pl program was used for the analysis.

## 3. Results

No deterioration was observed in any of the patients undergoing WBC procedures during the study.

### 3.1. Endothelium Parameters

**Figure 2:**
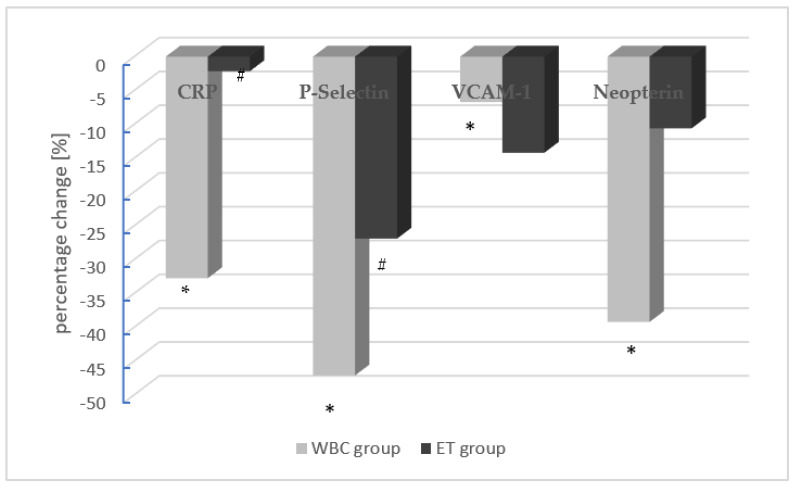
The mean percentage changes in endothelium function parameters in the WBC group (gray) and the ET group (black). The concentration of a given parameter at the first collection was assumed as 100%. Statistically significant differences between the first and second measurement (pre- and post-procedure) are marked as * and # in the WBC and ET groups, respectively (hsCRP: high-sensitivity C-reactive protein; sVCAM-1: soluble vascular cell adhesion molecule-1; (p): plasma; (s): serum).

AS patients in the WBC group exhibited a statistically significant decrease in the levels of hsCRP, sP-Selectin, sVCAM-1, and neopterin after the end of treatment. In the ET group, only the levels of sP-Selectin decreased significantly after the treatment. In turn, the levels of hsCR, sVCAM-1, and neopterin did not change significantly in the ET group, which underwent only exercise training after completion of the treatment (Table 2). The mean percentage changes in endothelium function parameters in the WBC group (gray) and the ET group (black) are also shown in Figure 2.

### 3.2. Oxidative Stress Parameters

**Figure 3:**
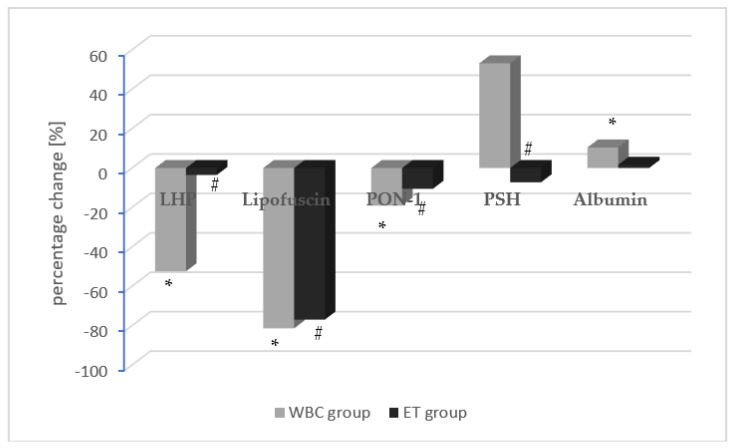
The mean percentage change in oxidative stress parameters in the WBC group (gray) and the ET group (black). The concentration of a given parameter at the first collection was assumed as 100%. Statistically significant differences between the first and second measurement (pre- and post-procedure) are marked as * and # in the WBC and ET groups, respectively (LHP: lipid hydroperoxide; PON-1: paraoxonase-1; PSH: protein sulfhydryl; (e): erythrocyte lysates; (p): plasma; (s): serum).

After the end of the treatment, the concentrations of LHP, lipofuscin, and PON-1 significantly decreased in both study groups, and the drop in these parameters was greater in the WBC group compared to the ET group. Moreover, after the treatment, the PSH level decreased significantly only in the ET group, but did not change significantly in the WBC group. Additionally, the albumin levels significantly increased in the WBC group, whereas they did not change after the treatments in the ET group (Table 3). The mean percentage change in oxidative stress parameters in the WBC group (gray) and the ET group (black) are also shown in Figure 3.

## 4. Discussion

An increasingly popular method of treatment for AS patients is WBC with subsequent exercise training, but little is known about the impact of this treatment on the endothelium. In our study, we observed that, after the treatment in the WBC group, the levels of hs-CRP, sVCAM-1, and neopterin decreased significantly compared to the ET group. The oxidative stress parameters (LHP, PON-1, and lipofuscin) and the level of sP-Selectin decreased in both studied groups, but the drop was greater in the WBC group compared to the ET group. In turn, the albumin level increased significantly after the treatment in the WBC group. 

In AS patients, chronic and systematic inflammation, impaired lipid metabolism, and enhanced lipid peroxidation can lead to endothelial dysfunction, which is characterized by a shift in the actions of the endothelium toward reduced vasodilation, a proinflammatory state, and prothrombic properties [11,30,31]. Increased levels of CRP in AS patients can decrease endothelial *nitric oxide synthase* (eNOS)-mediated nitric oxide (NO) production by decreasing the stability of eNOS mRNA. Thus, CRP, in addition to being a marker of an inflammatory state, may directly cause endothelial dysfunction [32]. As a consequence of these conditions, the endothelial phenotype changes to a proinflammatory and prothrombotic state through increased expression of leukocyte adhesion molecules (such as VCAM-1 and P-selectin) and cytokines such as monocyte chemoattractant protein-1. Levels of sVCAM-1 in plasma may increase with activation of the endothelial cells during systematic inflammation through several mediators, including reactive oxygen species [33,34,35]. It is the first adhesion molecule expressed before atherosclerotic plaque development [36]. Furthermore, endothelial P-selectin and VCAM-1 are also capable of supporting the adhesion of T cells under blood flow [37]. The above-described changes occurring during endothelial dysfunction enhance monocyte adhesion to and penetration through the vascular wall, playing a crucial role in atherosclerotic lesion formation [38].

Neopterin derived from guanosine triphosphate is a low-molecular-weight compound. The most important source of neopterin is human monocytes and macrophages stimulated by interferon-γ [39]. In AS patients, the serum level is considered of neopterin a marker of macrophage activation and disease activity [40]. Recently, it was shown that endothelial cells, not only monocytes and macrophages, are responsible for high serum neopterin levels, which are also known to synthesize neopterin [41]. Moreover, dysfunction of the endothelium and reduced arterial elasticity may be also connected with elevated levels of neopterin in plasma [42]. Additionally, in patients with carotid, cerebral, and coronary artery diseases, as well as aortic aneurysms, plasma concentrations of neopterin were higher. Furthermore, the advancement of coronary artery disease is positively correlated with increased levels of neopterin [43]. In turn, recent research conducted by Shirai et al. [44] reported that neopterin may have an atheroprotective effect. In endothelial cells, the expression of intercellular adhesion molecule-1 (ICAM-1), vascular cell adhesion molecule-1 (VCAM-1), and monocyte chemotactic protein-1 (MCP-1) is reduced by neopterin; thus, adherence of monocytes to the endothelium is decreased. Moreover, neopterin also reduces the inflammatory phenotype of monocyte-derived macrophages. Additionally, the migration and proliferation of vascular smooth muscle cells and the formation of foam cells incited by oxidized low-density lipoproteins in macrophages are decreased by neopterin. The authors postulated that increased levels of neopterin could be connected to a regulatory mechanism associated with NO synthase upregulation in macrophages to contribute tetrahydrobiopterin (BH4) for excessive output NO production stimulated by lowered endothelial NO synthase in the development of atherosclerosis process.

Unfortunately, we observed significant differences in neopterin serum concentration between WBC and ET groups. Aziz et al. [45] reported that intraindividual (CV_I_) and interindividual (CV_G_) coefficients of variation for serum neopterin level in men were 14.5% and 69.4%, respectively. The high CV_G_ value in connection with the low number of patients in both groups can explain the obtained results. In addition, taking into account the similar disease activity measured by BASDAI and BASFI indices, the difference in neopterin serum level between groups is probably accidental. 

The observed decrease in the serum CRP and neopterin concentrations, as well as the subsequent decrease in sP-Selectin and sVCAM-1 adhesive molecules may be connected to the increased activity of nitric oxide synthase [46], as well as the decrease in ox-LDL and other parameters of oxidative stress and inflammatory state after WBC treatment, as also shown in our previous papers [21,22]. After the completion of WBC in our research, in AS patients, the PON-1 activity decreased significantly. We observed a similar effect in healthy subjects who underwent WBC treatment [24]. After the WBC treatment, the decrease in PON1 activity could be related to the reduced oxidation of lipids on LDL particles, or the decrease in the accumulation of oxidized lipids on HDL particles [47]. Our previous studies showed a decrease in ox-LDL levels after the WBC treatment [48]. 

PON-1 is the enzyme involved in phospholipids hydroperoxide disposal, in addition to having thiolactonase activity and reducing local homocysteine thiolactone production in the subendothelial space and atherosclerotic lesions. We observed a decrease in the primary lipid peroxidation products (i.e., LHP in this study; ox-LDL and MDA/TBARS in our previous study [48]) and secondary oxidative stress markers such as lipofuscin, combined with normalization of elevated activity of PON-1. This process may be associated with the change in structure and biological function of high-density lipoproteins, which are the carriers of paraoxonases. 

The albumin concentration in serum increased significantly in the WBC group but did not change in the ET group. Although its expression may be downregulated in the case of systemic inflammation, serum albumin is a liver protein. Therefore, albumin is a negative acute-phase protein [49,50]. Therefore, the increased albumin levels after WBC treatments in our study may be related to decreased inflammation and oxidative stress, which was also observed in this study and reported in the previous ones [17,20,21,22,51,52,53,54]. Furthermore, albumin is the main plasma protein rich in sulfhydryl groups and provides nonenzymatic antioxidant defense outside the cells. Along with an increase in albumin concentration in the WBC group after therapy, we observed an increase in serum-free SH groups (PSH), which confirms our earlier observations of the beneficial effect of WBC on antioxidant status [21,22,48]. 

To the best of our knowledge, this study is the first to evaluate the effects of WBC treatments on adhesive molecules (soluble VCAM-1 and P-Selectin), neopterin, and albumin levels, as well as PON-1 activity, in patients with ankylosing spondylitis. Due to the fact that AS patients have increased CV mortality caused by endothelial dysfunction, the obtained results are very important for this group of patients. They show that WBC treatment may improve endothelial dysfunction in AS patients. However, it seems necessary to continue this research into the effects of WBC treatment on endothelium in patients with ankylosing spondylitis in order to obtain further evidence. 

In addition, the clinical state of patients with ankylosing spondylitis improved after WBC treatment. After the completion of the cycle of WBC procedures with subsequent exercise training, in the AS patients, we observed significant decreases in BASDAI and BASFI indices, as well as pain intensity, and an improvement in spine mobility parameters. In the WBC group with subsequent exercise training, we observed on average about twofold better results than in the group treated with only exercise training [19].

Our study had some limitations that should be acknowledged. Firstly, the study should have involved a greater number of AS patients to estimate the effect of size. Secondly, the WBC procedures only lasted 10 sessions, whereas a larger number could have increased the effectiveness of the treatment. Thirdly, we estimated the effect of WBC procedures directly after the 10 sessions of WBC procedures and did not provide a long follow-up. 

## 5. Conclusions

Whole-body cryotherapy treatment decreases inflammatory endothelium parameters in AS patients with the active phase of the disease and could decrease endothelium dysfunction in this group of patients. However, further studies should be conducted. 

## Figures and Tables

**Table 1:** Demographic data of the patients covered by the study.

Characteristic	WBC Group (n = 16)	Exercise Training (ET) Group (n = 16)	*p*-Value
Age, years, mean (SD)	46.63 ± 1.5	45.94 ± 1.24	0.114
Sex M/F	16/0	16/0	-
BMI, kg/m^2^, mean (SD)	24.24 ± 4.4	23.76 ± 6.8	0.880
BASDAI index	5.43 ±1.61	5.28 ± 1.71	0.720
BASFI index	5.20 ± 2.29	5.01 ± 2.06	1.00
Smoking (yes/no)	0/16	0/16	-
Medication			
NSAID (yes/no)	16/0	16/0	-
DMARD (yes/no)	0/16	0/16	-
Biological agents (yes/no)	0/16	0/16	-

M: male; F: female; SD: standard deviation; BMI: body mass index; BASDAI: Bath Ankylosing Spondylitis Diseases Activity Index; BASFI: Bath Ankylosing Spondylitis Functional Index; NSAID: nonsteroidal anti-inflammatory drug, DMARD: disease-modifying antirheumatic drug.

**Table 2:** Endothelium parameters (mean value ± standard deviation SD) in AS patients before and after the treatment in the studied groups.

Parameters		WBC Group	ET Group	*p*
hsCRP (s) (mg/dL)	before	17.4 ±15.8	13.9 ± 15.2	0.532
	after	11.7 ± 15.0	13.6 ± 16.2	0.730
	P*	**0.013**	0.623	
sP-Selectin (p) (ng/mL)	before	171 ± 85.4	197 ± 92.1	0.405
	after	90.4 ± 42.4	144 ± 103	0.067
	P*	**0.011**	**0.044**	
sVCAM-1 (p) (ng/mL)	before	923 ± 135	922 ± 349	0.843
	after	861 ± 102	791 ± 344	0.611
	P*	**0.001**	0.215	
Neopterin (s) (ng/mL)	before	9.05 ± 6.37	5.47 ± 3.39	0.063
	after	5.50 ± 3.31	4.89 ± 2.70	0.223
	P*	**0.039**	0.600	

WBC group: AS patients exposed to whole-body cryotherapy and exercise training; ET group: AS patients exposed only to exercise training; hsCRP: high-sensitivity C-reactive protein; sVCAM-1: soluble vascular cell adhesion molecule-1; P*: statistical significance of differences between values before and after treatment in particular groups of subjects; P: statistical significance of differences between both groups of subjects; (e): erythrocyte lysates; (p): plasma; (s): serum.

**Table 3:** Oxidative stress parameters (mean value ± standard deviation SD) in AS patients before and after the treatment in studied groups.

Parameters		WBC Group	ET Group	*p*
LHP (s) (μmol/L)	Before	19.9 ± 23.7	14.3 ± 6.8	0.957
	After	9.47 ± 17.0	13.8 ± 21.9	0.537
	P*	**0.001**	**0.008**	
Lipofuscin (e) (RF/gHb)	Before	225 ± 43.6	201 ± 36.9	0.101
	After	41.9 ± 16.5	46.4 ± 31.8	0.623
	P*	**<0.001**	**<0.001**	
PON-1 (s) (IU/L)	Before	163.3 ± 74.9	185.5 ± 85.6	0.441
	After	132.2 ± 71.0	166 ± 82.8	0.224
	P*	**0.015**	**0.03**	
PSH (s) (μmol/L)	Before	402.6 ± 91.7	393.2 ± 90.0	0.872
	After	616.5 ± 279.1	364.7 ± 28.4	0.339
	P*	0. 921	**0.017**	
Albumin (s) (g/L)	Before	38.93 ± 2.29	40.59 ± 2.32	0.060
	After	42.98 ± 2.13	41.26 ± 2.38	<0.001
	P*	**0.044**	0.756	

WBC group: AS patients exposed to whole-body cryotherapy and exercise training; ET group: AS patients exposed only to exercise training; LHP: lipid hydroperoxide; PON-1: paraoxonase-1; PSH: protein sulfhydryl; P*: statistical significance of differences between values before and after treatment in particular groups of subjects; P: statistical significance of differences between both groups of subjects; (e): erythrocyte lysates; (p): plasma; (s): serum.

## Data Availability

Data supporting reported results are available upon request from the corresponding author.
